# Severe Hypercalcemia Following Pembrolizumab Therapy: A Case Report and A Literature Review

**DOI:** 10.2174/0118715303409910250725043347

**Published:** 2025-07-31

**Authors:** Massimiliano Lazzaroni, Francesco Angelini, Rinaldo Guglielmi, Roberto Novizio, Anjali Iadevaia, Aikaterini Andreadi, Enrico Papini

**Affiliations:** 1 Department of Systems Medicine, Session of Endocrinology and Metabolic Diseases, University of Rome Tor Vergata, Rome, Italy;; 2 Regina Apostolorum Hospital, Medical Oncology Unit, Albano Laziale, Rome, Italy;; 3 Department of Endocrine and Metabolic Diseases, Regina Apostolorum Hospital, Albano Laziale, Rome, Italy;; 4 Department of Science of Nutrition, Metabolism, Aging and Gender-Related Disease, Catholic University of the Sacred Hearth, Rome, Italy

**Keywords:** Immune checkpoint inhibitor (ICI), pembrolizumab, immune-related adverse event (irAE), programmed death-1 (PD-1) inhibitor, hypercalcemia, colorectal adenocarcinoma, osteoclast activation

## Abstract

**Introduction/Background:**

Immune checkpoint inhibitors (ICIs) play a central role in advanced cancer treatment, but they are associated with immune-related adverse events (irAEs) that include two cases of hypercalcemia induced by the programmed death-1 (PD-1) inhibitor, pembrolizumab. We report a unique case of a colon cancer patient treated with pembrolizumab who acutely developed life-threatening hypercalcemia.

**Case Presentation:**

This case presents a seventy-five-year-old woman suffering from colon adenocarcinoma with liver metastasis undergoing pembrolizumab therapy. Shortly after its second administration, she developed severe hypercalcemia (21 mg/dL) with acute kidney failure. Serum intact parathyroid hormone (PTH), parathyroid hormone-related peptide (PTHrP), 25-OH vitamin D, and 1,25-OH vitamin D were suppressed while computerized tomography (CT) imaging ruled out relevant osteolytic or granulomatous lesions. Treatment included hydration, infusion of zoledronic acid, and high-dose glucocorticoids. The patient's serum calcium levels normalized, and her condition improved. Primary hyperparathyroidism, ectopic PTHrP secretion, and ectopic 25(OH)D-1-hydroxylase expression were ruled out by clinical and laboratory data. Notably, experimental models of PD-1/PD-L1 inhibition have demonstrated increased bone resorption. Although the absence of specific bone turnover markers and a recent 18FDG-PET/CT scan partly limits the understanding of the pathophysiological mechanism, immune-mediated osteoclast activation represents a potential pathophysiological mechanism of acute reversible hypercalcemia.

**Conclusion:**

The present case is unique due to its early onset, absence of calcitriol and PTHrP elevation, and rapid response to corticosteroids. Serum calcium should be assessed both before each ICI’s dose administration and throughout treatment in case of symptoms suspicious for hypercalcemia to prevent the onset and progression of this rare but critical irAE.

## 1 INTRODUCTION

Immune checkpoint inhibitors (ICIs) play a central role in advanced cancer treatment [1] and, among them, the programmed death-1 (PD-1) inhibitor pembrolizumab is widely used in clinical practice [2]. However, they are associated with a spectrum of immune-related adverse events (irAEs) induced by their complex mechanism of immune response modulation [3, 4]. Electrolyte imbalances and endocrine dysfunctions, such as thyroiditis, type 1 diabetes, and hypophysitis, are documented among the irAEs side-effects. Conversely, severe hypercalcemia is an unusual complication [5, 6] and its underlying pathophysiology remains poorly understood.

The development of hypercalcemia affects up to 30% of cancer patients at some point during their illness and requires the differential diagnosis between malignancy-related hypercalcemia and ICIs-associated irAE. Hypercalcemic conditions induced by ICIs have been documented [7, 8], but only two cases were directly associated with pembrolizumab through the 1,25-OH vitamin D-based mechanism reported for the other ICIs [9, 10].

Herein, we present a unique case of a colon cancer patient treated with pembrolizumab who acutely developed life-threatening hypercalcemia. This report draws attention to an acute and potentially severe complication of this treatment.

## 2 CASE PRESENTATION

Seventy-five-year-old patient was diagnosed with colorectal adenocarcinoma, grade 3 (ADC G3), pT4b, pN2a, M1 (hepatic localization), R0 resection. Molecular testing identified the tumor as KRAS mutant (Kirsten rat sarcoma virus gene G12xa mutation) and MSI-H (microsatellite instability – high), while BRAF (v-Raf murine sarcoma viral oncogene homolog B gene) and NRAS (neuroblastoma RAS viral oncogene homolog gene) were wild types. She started pembrolizumab 200 mg, which was clinically well tolerated, and underwent a second treatment 3 weeks later. The following day, she presented to the emergency department due to a syncopal episode. Her arterial blood gas analysis revealed metabolic alkalosis (pH 7.502, standard HCO_3_^−^ 33.7 mmol/L, pCO_2_ 43.8 mmHg), hypokalemia (2.1 mmol/L), and hypercalcemia (ionized calcium 2.28 mmol/L).

Laboratory findings confirmed the presence of severe hypercalcemia, hypokalemia, and initial impairment of renal function (reference ranges and serum dosages at peak presentation of hypercalcemia are summarized in Table **1**). The relationship between calcemia and creatinine trends is depicted in Fig. (**1**). A Computerized Tomography (CT) scan of the brain ruled out brain metastasis and intra- or extra-axial hemorrhages.

On the third day after the second ICI administration, despite abundant hydration combined with diuretic therapy, she became uncooperative and soporous. Hypercalcemia (21.0 mg/dL) reached its peak, and estimated glomerular filtration rate worsened (eGFR, 49 mL/min/1.73 m^2^). High-dose glucocorticoid therapy was initiated (dexamethasone, 8 mg/day), along with a single dose of IV zoledronic acid (4 mg) and IV potassium chloride supplementation. The course of calcium levels and the timing of pembrolizumab administration, zoledronic acid infusion, and high-dose glucocorticoid treatment are represented in Fig. (**2**).

The following day, the clinical condition was stable. Hematochemical analyses revealed low levels of intact parathyroid hormone (PTH, 18 pg/mL), 25-OH vitamin D deficiency (calcidiol, 5.5 ng/mL), high adrenocorticotropic hormone (ACTH, 101.5 pg/mL) and cortisol (80 µg/dL), central hypothyroidism [thyroid-stimulating hormone (TSH) 0.36 μIU/mL, free triiodothyronine (T3) 1.49 pg/mL, free thyroxine (T4) 10.4 pg/mL], and a renin-angiotensin-aldosterone system within normal limits (aldosterone 63.8 pg/mL, direct renin 11.2 μIU/mL, aldosterone/renin ratio 0.57 ng/dL/mIU/L). Parathyroid hormone-related peptide (PTHrP, 10 pg/mL) and 1,25-OH vitamin D (calcitriol, 5 pg/mL) were suppressed, while angiotensin-converting enzyme (ACE, 26 U/L) was within normal limits. Urine analysis showed absence of urinary sediment. These values are summarized in Table **1**.

Electrolyte imbalance remained critical: corrected calcemia 19.5 mg/dL and kalemia 2.6 mEq/L; serum osmolality (374 mOsm/L) and natremia (147 mEq/L) were slightly above the upper limit. Thus, IV dexamethasone (45 mg/24 hours) was administered, potassium canrenoate (100 mg/day) was added, and the daily dose of IV furosemide was doubled (40 mg). Within 2 days, a marked improvement in calcemia (15.4 mg/dL) and exacerbation of hypernatremia (160 mEq/L) and hypokalemia (2.4 mEq/L) were registered. Low-dose glucocorticoid therapy (dexamethasone, 4 mg/day) and infusion of hypotonic solution were started. During the following week, a progressive normalization of electrolyte levels was observed: calcemia (10.2 mg/dL) returned to normal values, as well as kalemia (4.8 mEq/L) and natremia (139 mEq/L). The patient progressively improved until discharge.

## 3 DISCUSSION

The occurrence of hypercalcemia in cancer patients receiving ICIs may be related to various pathophysiological mechanisms. Endocrine disorders (primary hyperparathyroidism, hyperthyroidism and adrenal insufficiency) are the prevalent causes of hypercalcemia in general population while in cancer patients the most frequent cause is malignancy-associated hypercalcemia [11, 12]. In the present case, PTH levels were at the lower limit, ruling out primary hyperparathyroidism, while hyperthyroidism and adrenal insufficiency were excluded by the evidence of mild central hypothyroidism and high ACTH and cortisol. Finally, familial hypocalciuric hypercalcemia was also ruled out.

Malignancy-associated hypercalcemia is commonly induced by direct bone osteolysis due to metastatic tumor cells [13], by ectopic PTHrP secretion, or by ectopic calcitriol production [11, 12]. However, in the present case, whole-body CT imaging showed no evidence of osteolytic metastases or concurrent myeloma, and the cancer histotype [14], together with normal PTHrP levels, ruled out humoral hypercalcemia of malignancy. Finally, the suppressed calcitriol levels and the exclusion of concurrent lymphoma made a calcitriol-associated mechanism unlikely.

Granulomatous disease occasionally causes hypercalcemia by inappropriate macrophage activation, mainly in association with PD-1/PD-L1 inhibitors, through the activity of ectopic 25(OH)D-1-hydroxylase expressed in M2-polarized macrophages (calcitriol-dependent mechanism) [7, 9]. However, suppressed calcitriol and ACE concentrations within the normal range, together with non-significant pulmonary findings, made sarcoidosis unlikely; in addition, a negative QuantiFERON Gold assay and the absence of fungal infection excluded a granulomatous cause. Also, no drugs associated with elevation of blood calcium, such as thiazide diuretics, lithium, and calcitriol analogs [9], were administered.

The PD-1 pathway, crucial for preserving self-tolerance, has been shown in experimental models [15] to also regulate osteoclastogenesis through a calcitriol-independent mechanism. Hence, ICIs’ interference may enhance osteoclast differentiation and activity, leading to increased bone resorption and subsequent hypercalcemia through an elevated RANKL/OPG (receptor activator of nuclear factor kappa-Β ligand/osteoprotegerin) ratio. This process is worsened by pro-inflammatory cytokines, such as TNF-α (tumor necrosis factor α) and IL-6 (interleukin 6), which lead to RANKL upregulation in osteoblasts, activated T cells, and synovial fibroblasts [16, 17]. To date, no large-scale systematic review nor clinical trial has reported hypercalcemia as a major irAE of pembrolizumab [18-20]. Few isolated case reports (Table **2**) have described pembrolizumab-associated hypercalcemia, all of which suggest a calcitriol-mediated mechanism. Notably, these cases were characterized by late occurrence (two years and nine months, respectively) of mild hypercalcemia [9, 21].

In the present case, the previously normocalcemic patient developed severe hypercalcemia shortly after the second administration of pembrolizumab. Biochemical findings, including suppressed serum PTHrP and calcitriol, together with PTH and ACE levels in the low-normal range, exclude the common mechanisms of malignancy-associated hypercalcemia and granulomatous inflammation. Additionally, mild central hypothyroidism and hypercortisolism rule out less frequent endocrine causes. Imaging showed no evidence of osteolytic metastases or granulomas, and the administered therapy did not include any drugs capable of causing hypercalcemia, apart from pembrolizumab itself. These findings suggest that immune-mediated osteoclast activation is the most plausible pathophysiological mechanism underlying this episode of acute, reversible hypercalcemia.

The limitations of the present report include the absence of specific bone turnover markers to confirm immune-mediated osteoclastic activation and the lack of bone-specific imaging, such as an 18FDG-PET/CT scan [22]. Furthermore, temporal relationship alone could be insufficient to establish causality between the second pembrolizumab administration and hypercalcemia, given the patient's advanced cancer status.

## CONCLUSION

The present case is unique for its early onset, absence of calcitriol elevation, and rapid response to corticosteroids. Early recognition and management of endocrine irAEs in patients receiving ICIs are crucial to minimize morbidity and maintain cancer treatment, especially in patients with elevated bone turnover markers.

Serum calcium should be assessed both before each ICI dose administration and throughout treatment if symptoms suspicious for hypercalcemia occur, to prevent the onset and progression of this rare but critical irAE.

## Figures and Tables

**Fig. (1):**
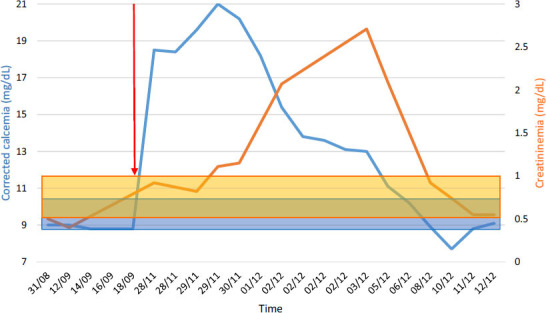
Corrected calcium levels (blue line) and creatinine trends (orange line) since the colon adenocarcinoma diagnosis. The blue and the orange areas represent the normal range of corrected calcemia and creatininemia, respectively. The arrow marks pembrolizumab administration.

**Fig. (2):**
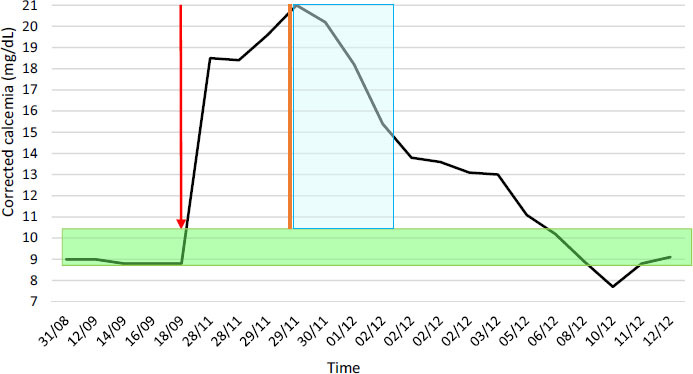
Corrected calcemia since colon adenocarcinoma diagnosis and timing of pembrolizumab administration (red arrow), zoledronic acid infusion (orange line) and high dose glucocorticoid treatment (blue area).

**Table 1:** Serum dosages at peak presentation of hypercalcemia.

-	**Result**	**Reference Range**
**Arterial Blood Gas Analysis**		
pH	7.502	7.35-7.45
Standard HCO_3_^-^ (mmol/L)	33.7	22-26
pCO_2_ (mmHg)	43.8	35-45
Potassium (mEq/L)	2.1	3.5-5.1
Ionized calcium (mg/dL)	9.13	4.5-5.3
**Hematochemical Analyses**		
Corrected calcium (mg/dL)	21	8.8-10.4
Calcium (mg/dL)	20.3	8.8-10.4
Phosphate (mg/dL)	5.1	2.5-5.2
Magnesium (mg/dL)	2.32	1.6-2.6
Sodium (mEq/L)	160	136-145
Potassium (mEq/L)	2.4	3.5-5.1
Intact PTH (pg/mL)	18	18.5-88
PTHrp (pg/mL)	10	8-20
25-OH vitamin D (ng/mL)	5.5	> 30
1,25-OH vitamin D (pg/mL)	< 5	19.9-79.3
Creatinine (mg/dL)	1.15	0.55-1.02
eGFR (mL/min/1.73 m^2^)	47	> 90
Serum osmolality (mOsm/L)	374	280-300
TSH (μIU/L)	0.36	0.48-4.17
Free T3 (pg/mL)	1.49	2.3-4.2
Free T4 (pg/mL)	10.4	8.9-17.6
Calcitonin (pg/mL)	4.24	1.0-4.8
Renin (μIU/mL)	11.2	2.8-39.9
Aldosterone (pg/mL)	63.8	11.7-236.0
ACE (U/L)	26	20-70
ACTH (pg/mL)	101.5	4.7-48.8
Cortisol (μg/dL)	> 80	4.5-24.0

**Table 2:** Summary of reported cases of pembrolizumab-associated hypercalcemia.

**Author**	**Year**	**Cancer Type**	**ICI Exposure Timing**	**Proposed Mechanism**	**Calcitriol**	**Treatment**	**Outcome**
Donangelo *et al*. [21]	2022	Endometrial cancer	9 months	Sarcoid-like granulomatosis	Increased	Steroids, calcitonin, bisphosphonate (zoledronic acid)	Recurrence post-rechallenge, then resolution
Oommen *et al*. [9]	2023	Bladder and prostate cancer	2 years	Macrophage-mediated calcitriol excess	Increased	Steroids, calcitonin, denosumab	Resolution, maintained
Present case	2025	Metastatic colon cancer (MSI-H, KRAS mutant)	Shortly after the 2^nd^ dose	Non-calcitriol-mediated, likely osteoclast activation	Normal	Steroids, bisphosphonate (zoledronic acid)	Rapid resolution

## Data Availability

Not applicable.

## LIST OF ABBREVIATIONS

ICIs: Immune Checkpoint Inhibitors
irAEs: Immune-Related Adverse Events
PD-1: Programmed Death-1
KRAS: Kirsten Rat Sarcoma Virus Gene G12X Mutation
MSI-H: Microsatellite Instability – High
BRAF: v-Raf Murine Sarcoma Viral Oncogene Homolog B Gene
NRAS: Neuroblastoma RAS Viral Oncogene Homolog Gene
eGFR: Estimated Glomerular Filtration Rate
PTH: Parathyroid Hormone
PTHrp: Parathyroid Hormone-Related Peptide
TSH: Thyroid-Stimulating Hormone
FT3: Free Triiodothyronine
FT4: Free Thyroxine
ACE: Angiotensin-Converting Enzyme
CT: Computed Tomography
RANKL: Receptor Activator of Nuclear Factor Kappa-Β Ligand
OPG: Osteoprotegerin
TRAP: Tartrate-Resistant Acid Phosphatase
TNF- α: Tumor Necrosis Factor α
IL-6: Interleukin 6

## STANDARDS OF REPORTING

CARE guidelines were followed.
